# Evaluating Strategies to Assess the Differentiation Potential of Human Pluripotent Stem Cells: A Review, Analysis and Call for Innovation

**DOI:** 10.1007/s12015-024-10793-5

**Published:** 2024-09-28

**Authors:** Lucy Smith, Rebecca Quelch-Cliffe, Felicity Liu, Alejandro Hidalgo Aguilar, Stefan Przyborski

**Affiliations:** 1 Department of Biosciences, Durham University, Durham, England; 2 Reprocell Europe Ltd, NETPark, Sedgefield, England

**Keywords:** Pluripotent stem cells, Pluripotency, Differentiation, Methods, Teratoma xenograft, Cell culture technology, In vitro

## Abstract

Pluripotent stem cells have the ability to differentiate into all cells and tissues within the human body, and as a result they are attractive resources for use in basic research, drug discovery and regenerative medicine. In order to successfully achieve this application, starting cell sources ideally require in-depth characterisation to confirm their pluripotent status and their ability to differentiate into tissues representative of the three developmental germ layers. Many different methods to assess potency are employed, each having its own distinct advantages and limitations. Some aspects of this characterisation process are not always well standardised, particularly techniques used to assess pluripotency as a function. In this article, we consider the methods used to establish cellular pluripotency and subsequently analyse characterisation data for over 1590 human pluripotent cell lines from publicly available repositories in the UK and USA. In particular, we focus on the teratoma xenograft assay, its use and protocols, demonstrating the level of variation and the frequency with which it is used. Finally, we reflect on the implications of the findings, and suggest *in vitro* alternatives using modern innovative technology as a way forward.

**Graphical Abstract:**

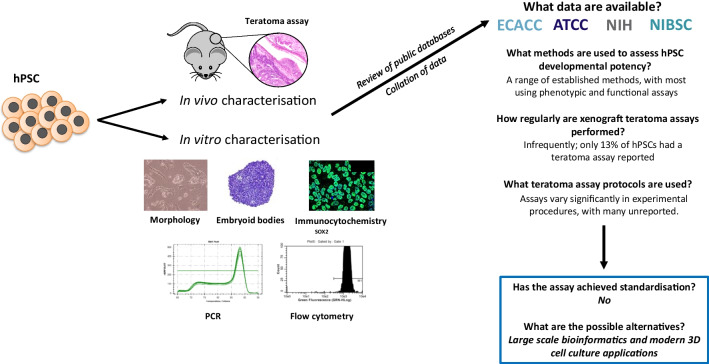

## Introduction

Pluripotency is defined as the ability of a cell population to self-renew and produce differentiated progeny derived from all three developmental germ layers; ectoderm, mesoderm and endoderm. Thorough confirmation of this property in stem cell lineages is crucial for their successful use in downstream applications, particularly in regenerative medicine and methods of tissue differentiation. This is especially pertinent when the variability in differentiation capacity of pluripotent stem cell (PSC) lines is considered [1], and is of importance when selecting a lineage for experimentation. Ideally the lineage should consist of a pure PSC population with the ability to generate a high yield of differentiated progeny that exhibit appropriate physiological function. Moreover, PSCs must be safe for use in clinical applications without risk of dedifferentiation or development of a malignant phenotype. Standardisation of the processes involved in PSC culture, including derivation, banking [2–4], characterisation [5], storage and maintenance [6] continues and will be imperative to the successful routine use of PSCs. However, consistently low yields, impurities and immature phenotypes from differentiation protocols can hinder the adoption of PSCs for routine use in the laboratory and prevent transition of potential therapies to the clinic. Such heterogeneity of PSC differentiation capacity is likely to be due to numerous diverse influences including genetic variation and microenvironmental effects within the culture dish [1, 7, 8].

**Fig. 1:**
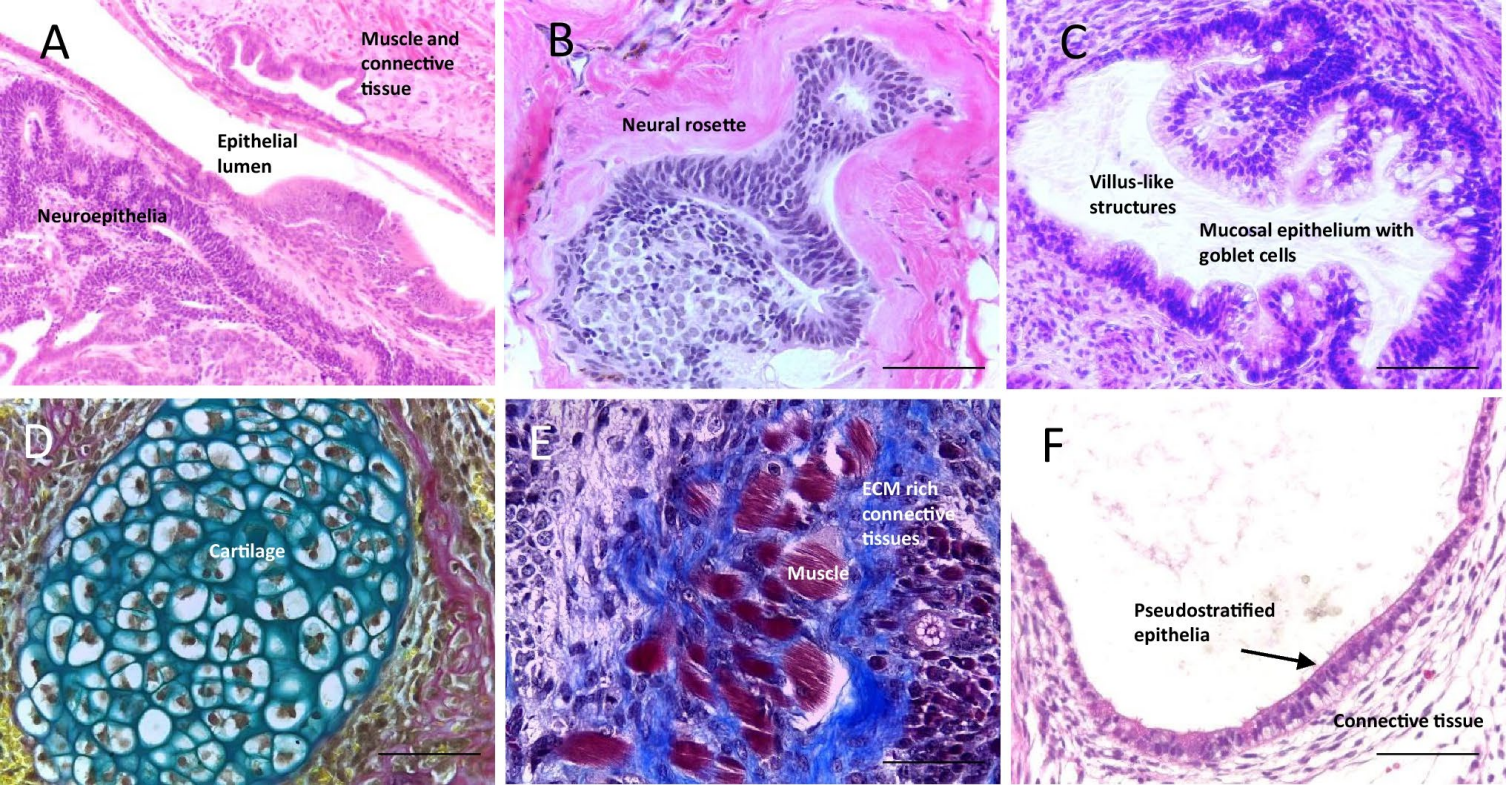
Representative differentiated tissue structures observed in xenograft teratomas formed from engraftment of human embryonic stem cells into an immune deficient mouse host: The identification of structures from the three germ layers within a xenograft teratoma via histological analysis is sufficient to confirm pluripotency in putative human stem cell populations. Images are Haematoxylin and Eosin (H&E) stained unless stated otherwise: **A**) Low magnification image of a typical teratoma, showing diversity of complex yet disorganised tissue structures from the three primary germ layers; **B**) Neuroepithelial tissues organised in a neural rosette structure; **C**) Intestinal epithelium structures with villus-like projections; **D**) Weighert’s staining highlighting cartilage tissue (blue) surrounded by rudimentary bone tissue (red); **E**) Masson’s Trichrome staining highlighting extracellular matrix rich areas (blue) surrounding striated muscle tissues (red); **F**) Pseudostratified epithelial lumen structure surrounded by varied connective tissues. Scale bars: 100 µm (**E**) and 50 µm (**B**, **C**, **D**, **F**)

Various methods have been developed to assess pluripotency, ranging from simple morphological analysis to complex animal experiments (see Tables 1 and 2). Each has its own advantages and limitations, according to the ease with which these assays can be performed and the data that can be generated. More specifically, these techniques can be further subdivided into those which assess pluripotency as a state, and those which assess pluripotency as a function. The importance of this distinction is clear when considering the fact that the identification of molecular pluripotency signatures which are commonly observed in pluripotent populations, i.e. identifying pluripotency as a state, does not necessarily provide any indication regarding the differentiation capacity, i.e. pluripotent function, of a given population or highlight subtle heterogeneities between different PSCs [1, 5, 9, 10]. This in itself can be an issue when selecting the appropriate PSC lineage. Accordingly, assays which assess pluripotency as a function (also termed developmental potency, differentiation potential or developmental/differentiation capacity) are imperative to the characterisation process and involve a wide range of *in vitro* and *in vivo* techniques. For many years, the classic teratoma xenograft assay has been considered the ‘gold standard’ method [11, 12]. The formation of highly complex, mature, morphologically identifiable tissues derived from the three germ layers is considered empirical proof of PSC differentiation capacity (see Fig. 1). Teratoma data has been regarded essential in the characterisation of new PSC lines, and has previously been endorsed by the International Stem Cell Banking Initiative [5].

**Table 1:** Methodologies used to assess and monitor human stem cell pluripotency: key aspects, advantages and disadvantages

Technique	Key aspects	Advantages	Disadvantages	References
Phase contrast microscopy	Cells grow in tightly packed colonies, with prominent nucleoli and high nuclear to cytoplasmic ratio	Rapid and inexpensive approach during routine culture and maintenance Distinctive morphology can indicate culture health in response to variable conditions	Limited information other than the characteristic structure of PSCs when observed in culture	[38, 39] [40]
Alkaline Phosphatase (AP) staining	Membrane bound enzyme is elevated in nearly all embryonic stem cell populations and decreases as cells differentiate. Simple detection by colorimetric assay	Expression mainly restricted to embryonic populations, making AP a sensitive marker Assays are rapid and inexpensive to perform, making it useful for monitoring cell status	Not completely exclusive to PSCs Provides limited information other than indication of PSC identity	[41] [42]
Immunocytochemistry	Antibodies detect the expression of key pluripotency associated markers, such as transcription factors Oct4, Sox2 and Nanog and extracellular membrane proteins such as SSEA-4 and TRA-1–60. Fluorescent or histochemical staining methods can be used	Can provide an overview of colony homogeneity and health Relatively inexpensive and accessible to most laboratories, useful for monitoring	Qualitative rather than quantitative Expression of markers in isolation does not indicate pluripotency, and there may be no correlation between markers Some markers not fully exclusive to PSCs	[1, 43–48]
Flow cytometry	Uses antibodies to detect multiple markers and sort subpopulations. Usually used to detect transcription factors and proteins associated with pluripotency	High throughput and rapid, the detection of multiple markers in the same population is useful for monitoring Quantitative, and gives an overview of an entire population, accounting for heterogeneity between colonies	Interpretation can be subjective Markers not fully exclusive and do not necessarily indicate pluripotent function	[49–51]
Karyotyping	Assesses the chromosome number and integrity of the PSC population. Specific stains are used to visualise the chromosomal banding patterns	Helps to detect aberrations/variation which could impact on functional pluripotency Useful for monitoring genomic integrity in response to culture conditions	Does not directly assess the pluripotency of stem cell populations	[52]
Epigenetic or transcriptome analysis	Analysis of epigenetic modifications within PSC genomes or RNA content in cells to determine which genes are actively being expressed under given conditions	Quantitative and can be performed on pluripotent and differentiated populations, (some assessment of pluripotent function) High throughput and can be combined with large datasets for increased accuracy and validation DNA methylation can be cell type specific, allowing for increased sensitivity. Single cell RNA-seq can provide insights into heterogeneity within a given sample	Can be difficult to delineate between mesoderm and endoderm populations Gene expression in the pluripotent state does not necessarily correlate with functional pluripotency Gene expression does not always correlate with downstream protein expression May not be able to detect subtle differences such as lineage biases	[31–33, 53–58]

**Table 2:** Methodologies used to assess and monitor the development potential of human stem cells: key aspects, advantages and disadvantages

Technique	Key aspects	Advantages	Disadvantages	References
Spontaneous differentiation	Removal of pluripotency maintenance conditions (such as feeder layers or growth factors) results in spontaneous differentiation of PSCs	Inexpensive, accessible and rapid during PSC culture, can be used to determine lineage biases Combination with quantitative methods can provide more conclusive data	Inherent simplicity will produce immature tissues or differentiation towards preferred lineages, not representing full differentiation capacity Culture conditions may influence differentiation and affect reproducibility	[39, 59, 60]
Directed differentiation	Addition of exogenous morphogens or chemicals to induce differentiation of PSCs toward certain phenotypes	Potential for highly controllable and directed differentiation into specific cell types, which is relatively inexpensive and accessible Can provide more conclusive data regarding differentiation when combined with quantitative methods	Inherently simple, may not represent full differentiation capacity and functional mature phenotypes may not be achieved Multiple additional factors absent that may influence cell differentiation (e.g. physical environment cues, timing etc.)	[61]
Embryoid body formation	Cells self-organise into spherical structures, often in suspension, following removal of pluripotency maintaining conditions, resulting in differentiation towards the primary germ layers	Many techniques to form EBs are accessible and inexpensive (e.g. hanging drop, low adhesion culture plates). Can be used for spontaneous or directed differentiation The presence of the three germ layers is more indicative of differentiation capacity, and can be combined with quantitative methods for more conclusive data	EBs are relatively immature structures, often with haphazard organisation. Considered by some not to be a stringent method of assessing pluripotency Formation of a hypoxic central core due to increasing EB size may impact differentiation and result in cell death. This may limit longer term studies	[12, 62–66]
Teratoma assay	Subcutaneous or internal implantation of PSCs into an immunodeficient mouse host, followed by an extended growth period, enables formation of a benign differentiated tumour that may contain rudimentary tissue derivatives representative of all three germ layers	Can provide conclusive proof regarding the ability to differentiate into varied, complex, morphologically recognisable tissues Simple criteria; the presence of tissue from the three germ layers confirms pluripotency Recognised, established technique that also provides data regarding malignancy (valuable for regenerative medicine)	Primarily qualitative morphological data and with inter tumour heterogeneity Labour intensive, time consuming and expensive (animal care and maintenance). Use of animals raises ethical issues Protocol variation between laboratories, known to impact on tumour differentiation. Few reporting standards	[11–13, 24–27, 29, 40, 67–73]
Modern 3D cell culture technology	Use of a combination of directed chemical cues and 3D culture techniques resulting in the differentiation of PSCs towards specific tissues or tissue rudiments	Can be highly customised to the tissue required and resultant structures can be analysed using standard techniques Morphologically identifiable tissues representative of each germ layer can be generated with a greater degree of control Avoids the need for animal resources	Requires technical skill to optimise growth conditions to direct cell differentiation Can be expensive due to the need for more specialised equipment and reagents Currently limited use for pluripotency, with few examples in the literature	[35–37, 74–85]

The teratoma xenograft assay is also considered the most rigorous method of confirming the pluripotency of human PSCs [12, 13], and generally, involves the implantation of an undifferentiated putative PSC population into a subcutaneous or internal location in an immunocompromised murine host. The subsequent formation of a tumour with evidence of tissues derived from each the three primary germ layers is indicative of pluripotency and assay success (See Fig. 1). Tetraploid complementation assays [14] and germ line transmission [15] are used to confirm developmental potential in non-human PSCs; these techniques can assess pluripotency even more comprehensively by demonstrating ability to direct differentiation for the formation of a fully competent organism capable of reproduction. Given that these methods involve the formation of chimeras, they cannot be used to characterise human stem cell lineages due to obvious ethical and legal restrictions.

The teratoma xenograft assay also has applications in developmental biology/organogenesis [16–20], cancer research [21], the study of pluripotency [22] and is considered to provide a wealth of data beyond the mere ability of PSCs to form the three germ layers. But while the teratoma assay is highly useful, it does have limitations. The need for a mouse host to conduct the assay puts it at odds with the now widespread practice of reducing animal use in research [23], and experimental success is not guaranteed. Variability in protocols and reporting is well recognised, however the scope and effects of this may be under appreciated, as these severely compromise data comparability and transparency, bringing into question the capability of the PSCs under analysis. In addition, many of these variable parameters influence differentiation trajectory [24–27]. The need for assay standardisation has been acknowledged by many [11, 12, 28], and while some attempts have been made [29, 30], these have not yet been widely adopted by the field.

There has been some drive to develop novel methodologies to assess the pluripotency, differentiation capacity and malignancy of PSCs. PluriTest is perhaps the most well-known, using an open access gene expression database of known PSCs to which microarray data from putative PSCs can be compared to assess pluripotency and any technical or biological variation between lineages [31, 32]. While these bioinformatics methods may provide strength of accuracy through use of large datasets, there are concerns regarding the use of a limited set of markers and the ability to detect subtle differences in lineage biases [33]. There have also been attempts to develop novel *in vitro* techniques to assess pluripotency, driven by advances in cell culture technology, the need to reduce animal usage, and to improve experimental accuracy, consistency and reproducibility. The ability to form complex tissue-like structures *in vitro* has been revolutionised through the development of technology that enhances the cellular microenvironment, including using three-dimensional culture systems such as physical scaffolds and hydrogels, perfused cultures and co-culture with other cell populations [34–37]. These methods can better recapitulate aspects and cues of the *in vivo* microenvironment, improving the ability to form tissue structures *in vitro*. This pursuit of complexity and consistency may resolve some of the issues associated with the teratoma xenograft assay and provide standardised and reproducible *in vitro* methods to investigate and confirm the differentiation capacity of PSCs.

In this article, we assess original characterisation data for human PSC lines registered for use at major UK and USA repositories in order to investigate trends in the methods employed to characterise the differentiation potential of PSCs. In particular, we focus on the combination of methods used to determine pluripotency and the frequency and reporting of the teratoma xenograft assay. While the lack of standardisation in assay reporting and performance continues to be appreciated [12, 28], it is of utmost importance to continue to challenge the currently accepted process. By assessing whether the methods used to characterise novel PSCs are suitable and sufficient, the scientific community as a whole can determine whether recommended practices are being adopted, if the teratoma xenograft assay can continue to be regarded as a ‘gold standard’ method and ultimately, innovate the most suitable methods to conclude whether a lineage in question is truly pluripotent.

## Materials and Methods

### Literature Search and Analysis

#### Research Strategy and Aims

An overall research strategy was determined and aims identified prior to commencing the literature search. Our assessment focus on the availability and characterisation processes used on human PSCs deposited at major cell banks in the United Kingdom (UK) and the United States of America (USA). This represents a significant body of data and much is freely available and readily accessible as a consequence of cell lineages being available for public use.

The aims of this literature review were to determine:

1. Whether all cell lines had characterisation data available for inspection.
2. What specific methods were used to assess pluripotency within the cell lines upon derivation.
3. Whether a teratoma xenograft assay had been performed.
4. If the teratoma assay had been performed, what parameters had been used and how had the assay been reported.

#### Study Selection

Major UK cell banks holding human PSCs were identified as: the National Institute for Biological Standards and Control (NIBSC) Research Grade Stem Cell Catalogue and the European Collection of Authenticated Cell Cultures (ECACC). Major US cell banks holding human PSCs were identified as: National Institutes for Health (NIH) Embryonic Stem Cell Registry and the American Type Culture Collection (ATCC).

For all repositories, complete lists of all human embryonic and induced PSCs were obtained from the online catalogues and collated, resulting in a total number of 1790 cell lines at the time of data collection. Lists were checked and duplicated lines removed, giving a final number of 1590 cell lines for analysis.

#### Data Acquisition and Analysis

Extensive literature searches were carried out to acquire the characterisation data for the human PSC lines. For each cell line, the aim was to find the original derivation and characterisation information in a journal article or failing that, to use the data from characterisation performed by the cell bank. Research included specific searches for each named cell line on hpscreg.eu and Cellosaurus to identify depositors and whether direct links to original articles could be found. Following this, individual searches were undertaken for each cell line in PubMed, Science Direct and Google Search to locate relevant information. For cell lines where no information could be found, original depositors were contacted to request the necessary data or papers.

#### Characterisation Analysis

Characterisation methods were split into two groups, pluripotent state and pluripotent function, depending on the aspect of pluripotency the technique assessed. Pluripotent state analyses were defined as any technique which assessed the presence of specific proteins or genes associated with pluripotency and included immunocytochemistry, flow cytometry, qPCR, alkaline phosphatase assay, karyotypic analysis and bioinformatic assessments such as PluriTest. Pluripotent function analyses were defined as any technique which involved in assessing the differentiation potential of pluripotent cells and included spontaneous differentiation (2D), embryoid body formation, directed differentiation (2D and 3D methods) and the teratoma xenograft assay.

#### Teratoma Parameter Analysis

Key assay parameters, many of which have been previously shown to impact on teratoma formation, were chosen and compared between cell lines. These were: number of cells transplanted, anatomical location and details concerning the murine host. Assay endpoint and success rate were also determined to be key parameters that could influence the interpretation of tumour growth.

#### Data Presentation

Microsoft Excel 2019 was used to collate and analyse the data, GraphPad Prism 5 was used for data visualisation.

### Generation of *In Vivo *and *In Vitro* Samples

Examples of data shared in this review have been generated from analysis of tissue blocks from previously published work [35]. Briefly, the following methods were used to generate these materials:

- *PSC cell culture:* Human H9 embryonic stem cells were used for this work. Cells maintained in feeder free conditions in 6 well plates (Greiner Bio One, Stonehouse, UK) coated with Matrigel hESC qualified matrix (Corning, Flintshire, UK) in mTESR plus medium (Stem Cell Technologies, Cambridge, UK) prepared according to the manufacturer’s instructions, as previously described in [35].
- *Teratoma xenograft assay:* H9 cells were maintained in feeder free conditions as described above, until required for the assay. Cells were detached from culture conditions using 0.25% trypsin/ 2mM EDTA (Fisher Scientific, Loughborough, UK) and counted using the Trypan Blue Exclusion Assay to obtain viable cell numbers. The teratoma assay was then performed as described in [35]. All procedures were completed under licence and permission according to the guidelines of the Home Office, United Kingdom.
- *Use of advanced 3D cell culture technology to assess differentiation potential of PSCs:* H9 cells were routinely maintained in feeder free conditions before cells were detached from plates using ReLeSR (Stem Cell Technologies), using ROCK inhibitor to enable the survival of cells in a single cell suspension. 3D tissue models were generated as described in [35].

## Results

### Characterisation Data was Available for more than 80% of PSC Lines at Public Repositories, yet those Lacking were Exclusively Embryonic Stem Cells

**Fig. 2:**
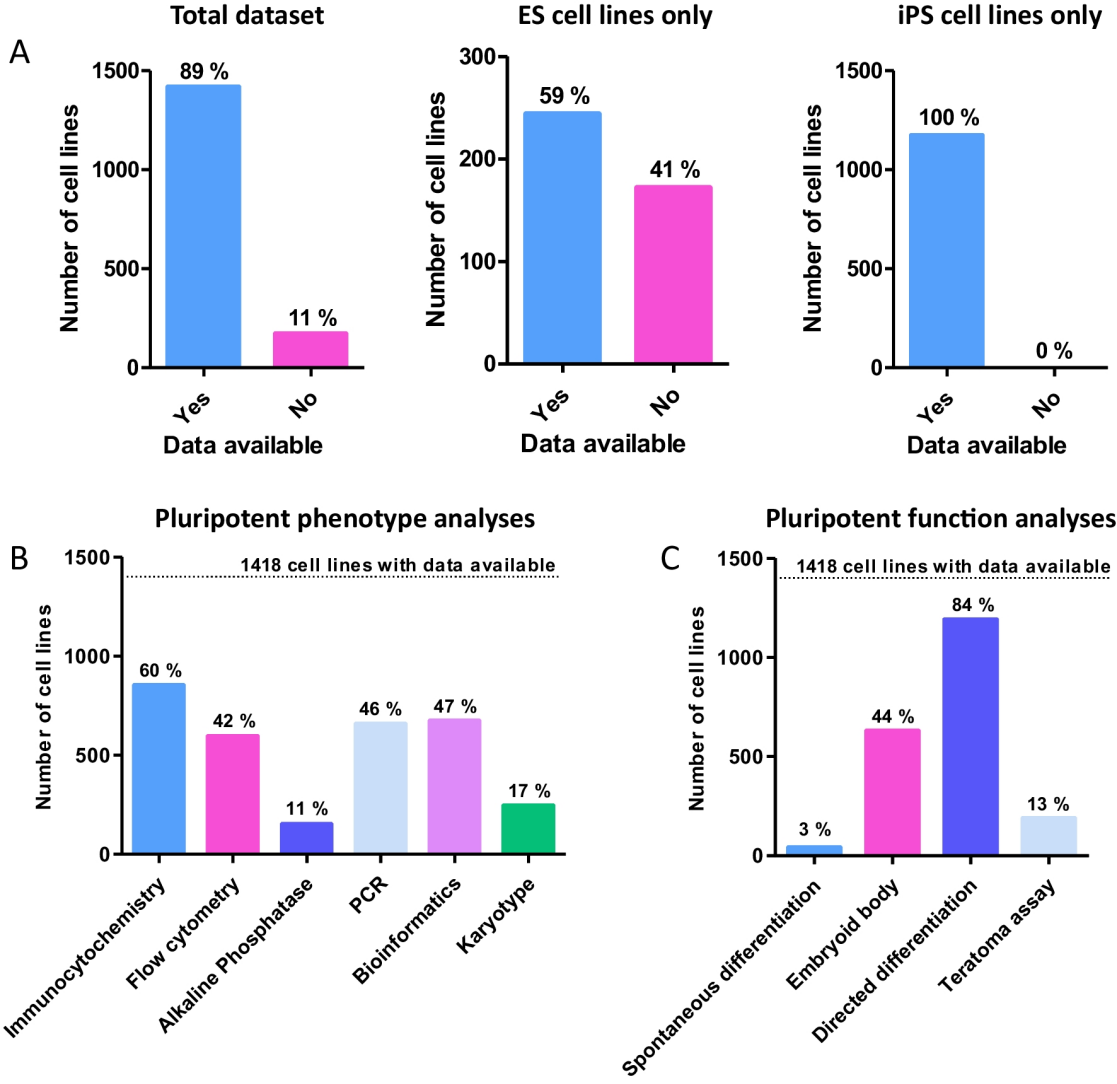
Analysis of characterisation data availability for hPSC cell lines and breakdown of the techniques used to determine pluripotency: Initial analyses focused on the availability of data in relation to cell line type as well as the techniques used to characterise hPSCs. **A**) Data availability for cell lines as compared to the entire dataset. Almost 90% of cell lines had some accessible characterisation data. A significant proportion of ES cells did not have any characterisation data available, 172 out of a total of 412 ES cell lines in the study. Some form of characterisation data was found for all 1174 iPSC lines surveyed. **B**) Breakdown of phenotypic analyses used to characterise hPSC lines. Phenotypic analyses had a smaller range of percentages, indicating diversity of methods available. Only immunocytochemistry was used on more than half of the cell lines surveyed, likely due to its simplicity and accessibility. **C**) Breakdown of functional approaches used to characterise hPSC lines. The use of functional analyses varied considerably depending on the technique. Directed differentiation (2D or 3D) was overwhelmingly the most popular in either category, used for 84% of cell lines (1191 total). This fits with the hypothesis that many PSC lines are derived for a specific purpose or study, but may be skewed by iPSCs derived in large scale studies and the high throughput nature of the technique

During the data collection phase, human PSC lines listed on the major UK and US repositories and cell banks were collated. Following the exclusion of duplicated cell lines, a total of 1590 original cell lines were identified as eligible for assessment. Each cell line was then extensively researched using a number of online resources in order to obtain the initial characterisation data. Original depositors were also contacted for cell lines where characterisation data was more difficult to source. In the event that initial characterisation data was unavailable, characterisation performed by the repository or cell bank was used for the analysis. This mainly applied to induced pluripotent stem cells (iPSCs), which had been derived on a larger scale as part of two projects by the cell banks themselves (EBiSC and HiPSC) (1,129 total cell lines). Of the 1590 lineages identified, 89% (1418) had basic characterisation data pertaining to pluripotency assessment available for analysis, whereas 11% did not have any characterisation data available from the primary literature or from initial analyses conducted by the cell bank (Fig. 2A). This proportion of lineages lacking data were exclusively embryonic stem (ES) cell lines. When this is considered as a fraction of the embryonic stem cell lines assessed in the study, the percentage without characterisation data is much higher, at 41% (Fig. 2A).

### Nearly all Cell Lines with Characterisation Data had at Least One Phenotypic and One Functional Assessment Performed

**Fig. 3:**
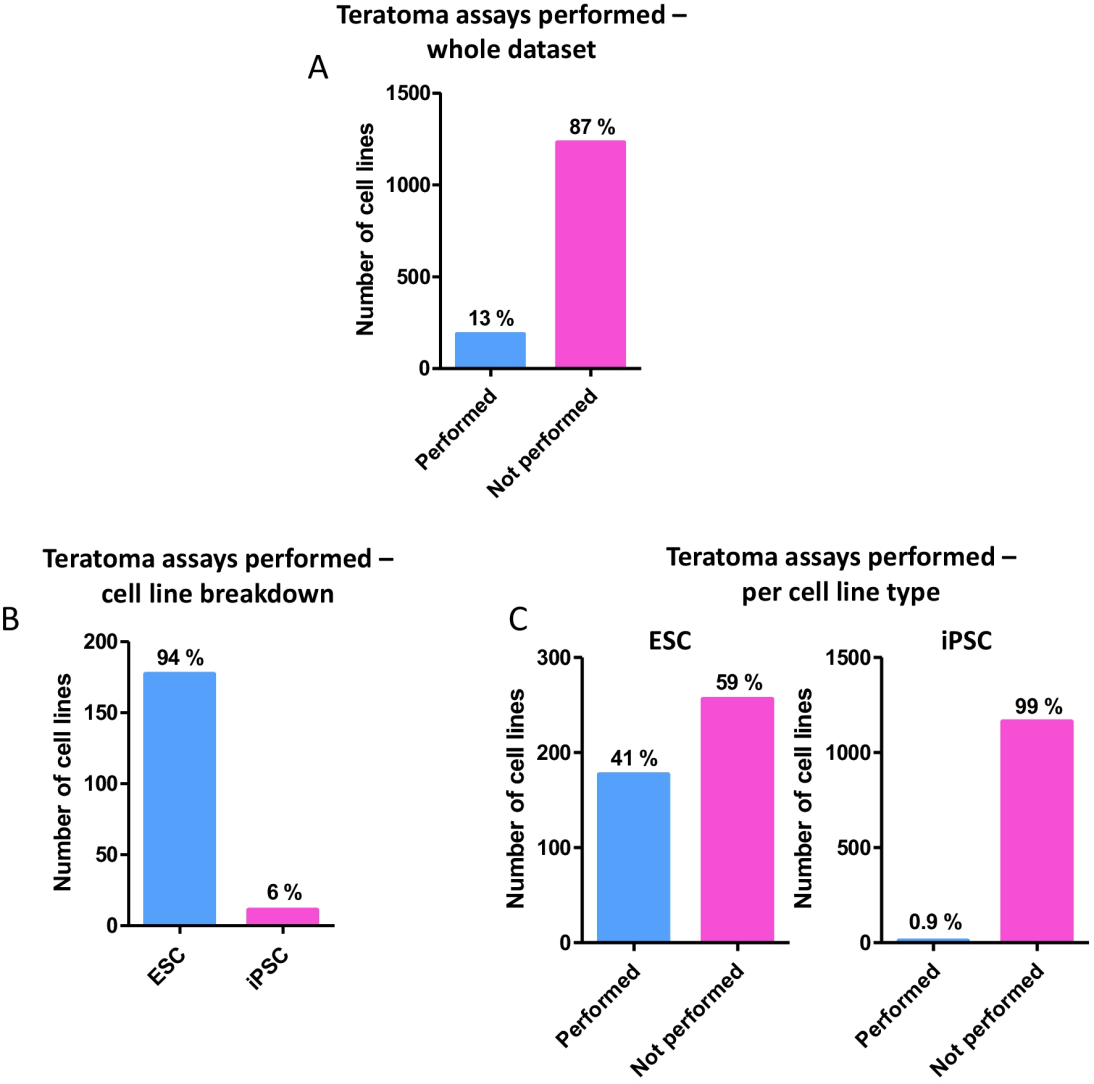
Evaluation of frequency of teratoma assay performance and analysis of PSC types on which the assay is performed: Analysis of teratoma assay use was performed in relation to data accessibility and each hPSC type. **A**) Percentage of registered human PSCs which had a teratoma assay performed, as a proportion all cell lines with characterisation data available. Only 13% of PSC lineages with accessible data had pluripotency confirmed using the teratoma assay, equating to 184 out of a total of 1418 cell lines. **B**) Of those cell lines that had the assay performed, 94% were ESC lines, whereas only 6% were iPSC lines. **C**) Percentage teratoma assays performed according to cell line type. For both ESCs and iPSCs, more cell lines did not have the teratoma assay performed that those that did. The difference is perhaps more surprising for ESCs which are generally derived in much smaller studies than iPSCs are (and with the associated skewing from the larger datasets)

Of the PSCs for which data could be obtained, 98% of cell lines had at least one phenotypic and one functional assessment performed. Of those methods considered to assess the pluripotent phenotype (immunocytochemistry, flow cytometry and AP assay), immunocytochemistry was overwhelmingly the most popular method with 60% of lineages having had the analysis completed (Fig. 2B). This was followed relatively closely by flow cytometry, at 42%. Performance of the AP assay appeared low, and this was mainly skewed by its use with iPSC lines (although only 55% of ES cells lines had the AP assay performed). Of the functional analyses assessing developmental potential, specific directed differentiation was the most popular method, with around 84% of the cell lines having had this completed (Fig. 2C). These methods were varied and included differentiation into cardiomyocytes, neurones and trophoblast lineages, as well as more general germ layer differentiation. In contrast, only 13% of the cell lines assessed had a teratoma xenograft assay performed (Figs. 2C, 3A). Of those that had the teratoma assay performed, 94% were ES cell lines, whereas only 6% were iPS cell lines (Fig. 3B). Of the ESC lines analysed just under half (41%, Fig. 3C) of ESCs had a teratoma assay performed, whilst of the 1174 iPSC lines analysed in this study an overwhelmingly high 99% had not been characterised using the method.

### Teratoma Assay Protocols Continue to Lack Standardisation, with the Most Common Response Being that Parameters and Procedures are Unreported

**Fig. 4:**
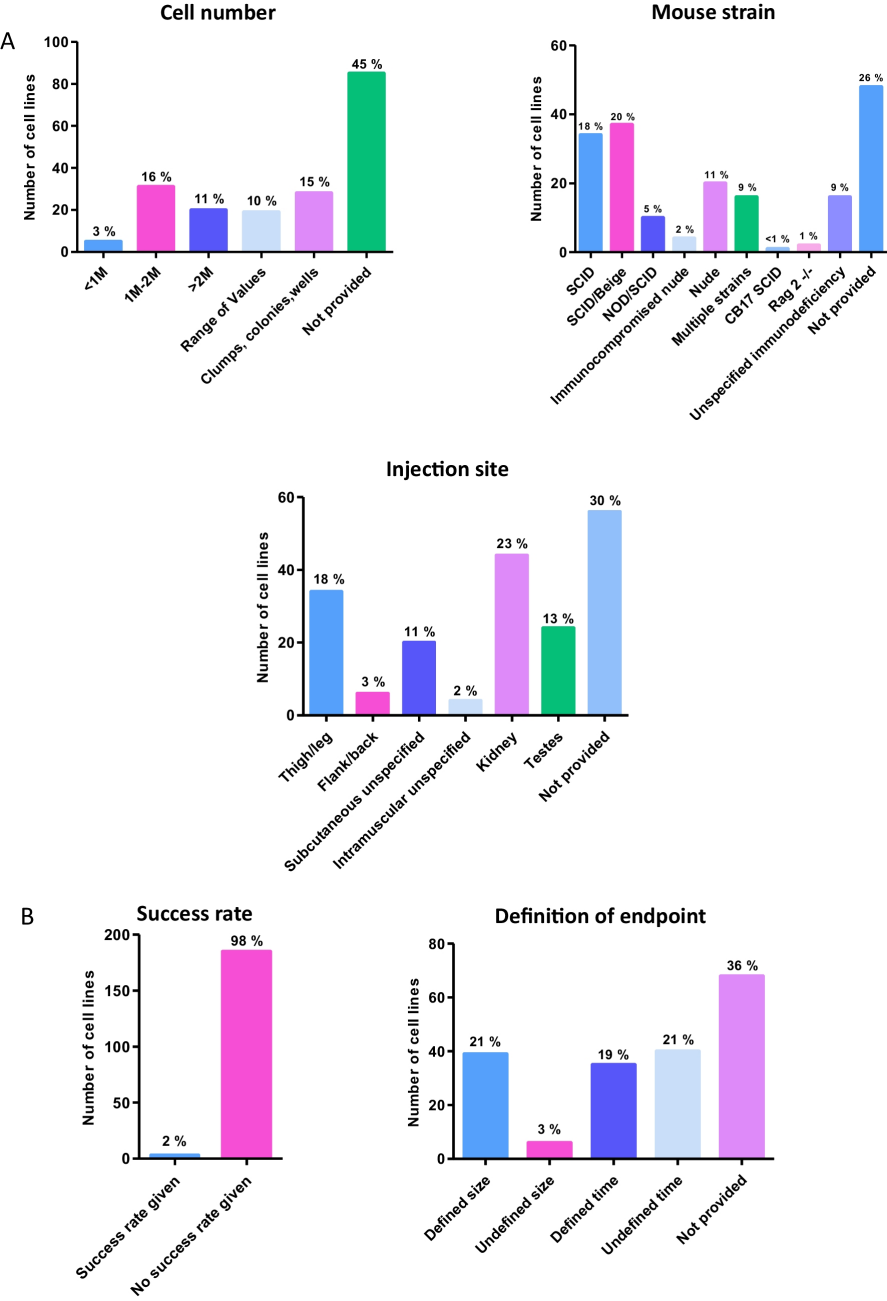
Analysis of the key experimental parameters and definitive aspects of the teratoma xenograft assay: In depth analysis of key teratoma assay protocol parameters provides additional useful data. **A**) Assay protocol parameters: Around 30% of assays provided specific data on the number of cells injected. Multiple studies used vague references such as clumps/wells/colonies, or a range of values. While SCID and SCID/Beige mouse strains were the most popular, a wide range of immuodeficient mouse hosts were used in this selection of teratoma assays, yet most frequently this information was not provided. Analysis showed some preference for thigh/leg or kidney injection sites, with a small number of studies not specifying the exact location but providing some information (subcutaneous or intramuscular). Most frequently the information concerning injection site was not provided. **B**) Assay completion parameters: Success rates can provide data on the effectiveness of protocols, although fear of judgement may prevent some from publishing this data. Of the 188 teratoma assays performed, only 3 provided information on teratoma formation success rates. Xenograft experiments may be brought to an end based on certain criteria. Such criteria could impact on assay results, and variability in this may prevent comparison. More assays used a defined endpoint than did not, but the modal response was that again the information was not provided

Deeper analysis into the protocols used to conduct the teratoma xenograft assay revealed a lack of congruency in this data subset. A number of key protocol parameters were assessed in depth (Fig. 4). Initial cell seeding number varied (Fig. 4A); some papers cited the use of a single cell suspension without a specific cell number, many papers used a specific initial cell seeding number but these ranged from less than 1 million to over 5 million cells per injection. A number of protocols used a range of values in the same assay set up, whereas some used highly arbitrary measures such as cell clumps or wells of a plate. Most commonly however, the number of cells used in the protocol was not specified (45% of 188 cell lines which had the assay performed).

The mouse strain used and anatomical site for injection for the teratoma xenograft were also assessed (Fig. 4A). Seven different immunodeficient mouse strains were used in the protocols analysed. In addition, some studies used multiple different immunodeficient mouse models as tumour hosts. While SCID/Beige was the most frequently used (20%), very closely followed by SCID (18%), around one third of the studies either did not detail the specific immunodeficiency of the mouse or did not provide any data about the mouse population used. As for the anatomical site where the PSC population was implanted, the use of renal (23%) and thigh/leg (18%) sites for injection was relatively common, but once again the modal response was that the anatomical site was not specified within the protocol (30% of 188 cell lines).

Information concerning tumour formation success rate and definition of the assay endpoint are also found to be generally lacking. Success rate was not provided for all but three of the cell lines assessed (Fig. 4B). Definition of the assay endpoint was broadly split into two categories, those based on the size of the tumour, and those based on time the tumour was left to grow but again, the manner in which the endpoint of the assay was determined was not provided for many of these studies (36%) (Fig. 4B).

### Alternative *In Vitro* Methods can Achieve Similar Levels of Tissue Complexity and Diversity to that Observed in the *In Vivo* Teratoma Assay

**Fig. 5:**
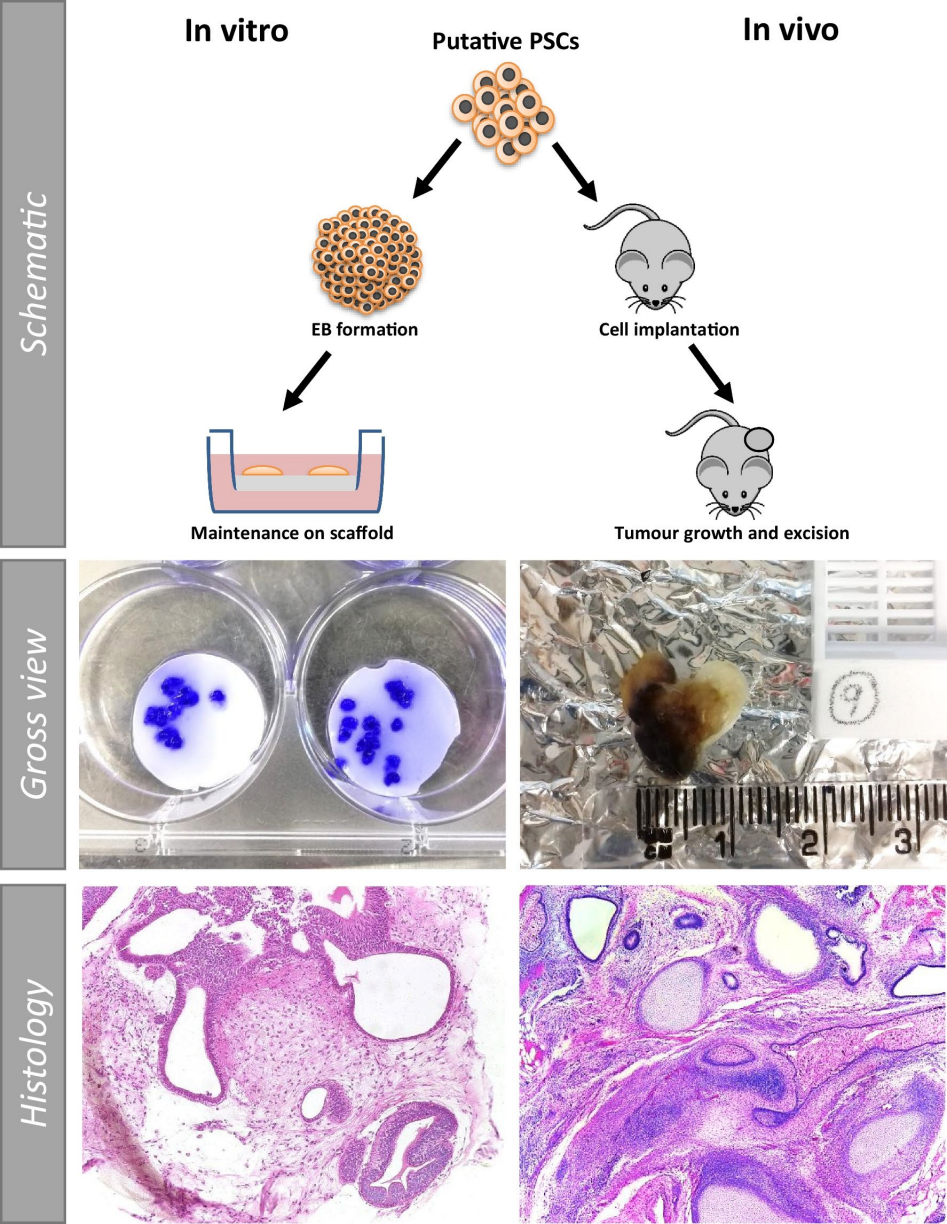
Schematic of technique comparison of *in vitro* vs *in vivo* structures derived from human pluripotent stem cells using either method: The *in vitro* method has a number of advantages, including the ability to generate multiple 3D teratoma structures from the same cell population in a single experiment, allowing for the assessment of a wider sample set. The gross view images of *in vitro* preparations (left), show individual crystal violet stained mini teratoma models. Low magnification histological staining highlights the diverse tissue structures which form using both techniques, demonstrating that teratoma structures exhibiting tissue complexity and diversity can be achieved utilising either *in vitro* or *in vivo* techniques

**Fig. 6:**
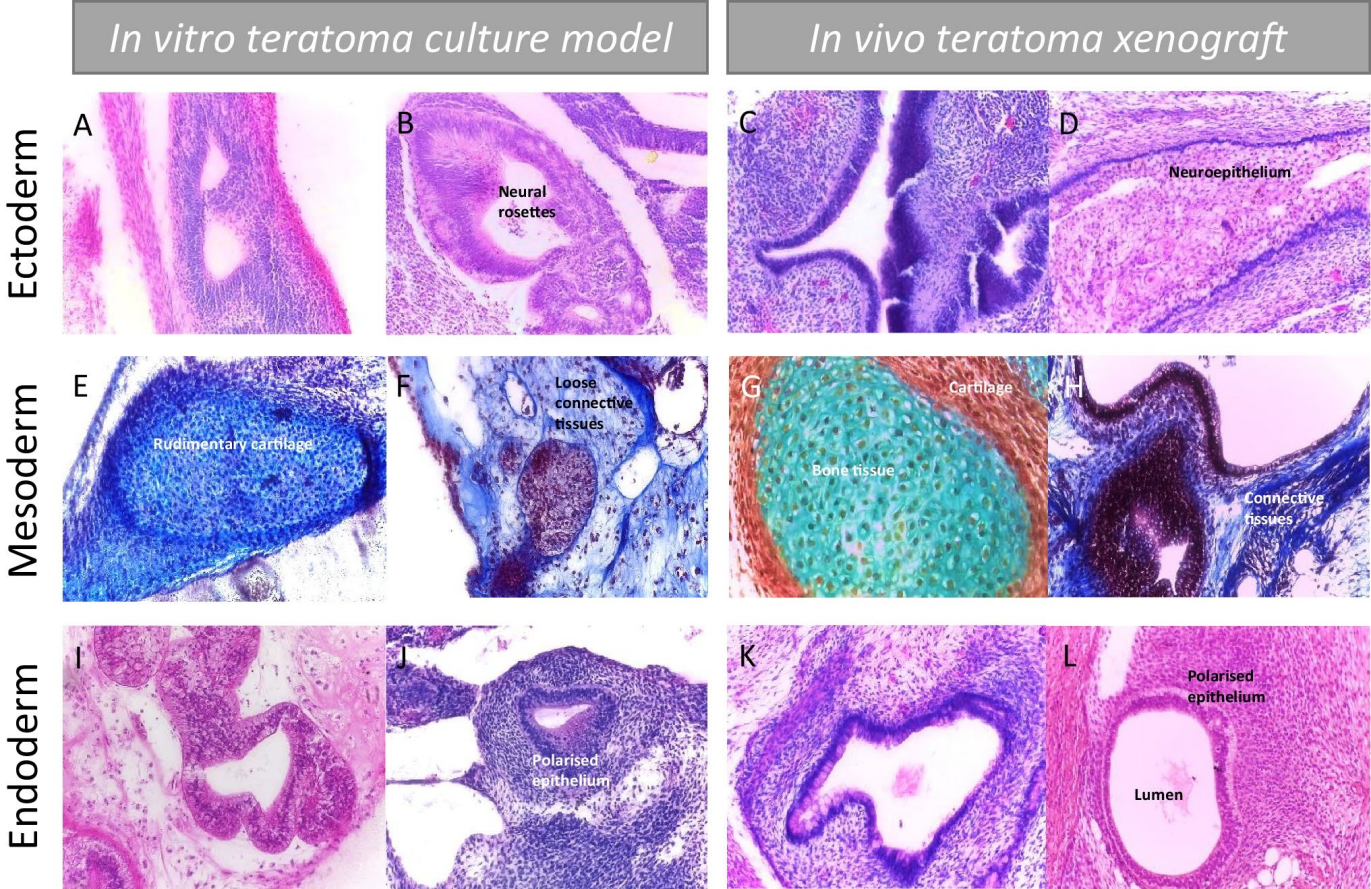
Assessment of functional pluripotency by teratoma tissue structures representative of different germ layers derived from hPSCs using either *in vitro* or *in vivo* techniques: Side by side histological comparison of samples from the *in vitro* and *in vivo* techniques shows the similarity in the tissue structures which form. Importantly, all three developmental germ layers can be identified in the *in vitro* sample, validating it against the simple criteria of the *in vivo* assay as a method for assessing functional pluripotency. **A**-**D**) H&E staining highlights neural rosettes and neuroepithelial structures in both sample types, confirming the presence of ectodermal derivatives. **E**–**H**) Masson’s Trichrome (MT) staining (**E**, **F**, **H**) and Weighert’s (WG) staining (**G**) indicate the structure and identity of the mesodermal structures within each sample type. Complex fibroblast derived extracellular matrix is demonstrated by blue MT staining of connective tissues, with evidence of diverse matrices noted in images **E** and **F**. Blue WG stain in image G highlights bone formation, surrounded by cartilage in red – rudimentary structures of this can be seen in the *in vitro* images. **I**-**L**) H&E staining clearly highlights the epithelial structures within the two samples. Polarised, organised cells surrounding a central luminal space can be observed across the samples, demonstrating the complexity of the tissue structures present

The ‘gold standard’ status of the teratoma assay is largely due to the fact that the complex, mature nature and diverse range of tissues found in successful teratomas has not previously been replicated *in vitro*. Previous work in our group [35, 37] has shown that tissue complexity similar to that observed in a teratoma can be achieved by combining EB formation with a porous scaffold (Fig. 5). Multiple small teratoma-like structures can be formed on the surface of the scaffold, with H&E staining demonstrating the varied nature of the *in vitro* structures in comparison to *in vivo* teratomas. Evidence of tissues derived from the three developmental germ layers, the key criterion for determining teratoma success, can be clearly observed in the *in vitro* teratoma model (Fig. 6). Highly similar structures can be produced using both experimental methods, as demonstrated by histological analysis.

## Discussion

Two previous studies have been performed to assess the characterisation of PSCs and the use of the teratoma xenograft assay within the stem cell field. In 2010, Müller and colleagues focused very specifically on whether the teratoma assay was used in articles which described the establishment of novel PSC lines [12]. While the study clearly argued that standardising the way the assay was performed and reported was essential to its successful use, and commented on protocols being ‘poorly reported’, much of the evidence was anecdotal, therefore not revealing the true extent of the variability issue. More recently, Montilla-Rojo published a review examining the teratoma xenograft assay as a tool to investigate both pluripotency and malignancy, providing an assessment of parameters used and considering the transparency of reporting in light of the ARRIVE guidelines for the use of animals in research [28, 86]. While this study does demonstrate the issues surrounding protocol variability, ultimately it focuses on articles which described use of a teratoma assay, therefore providing little information as to the frequency of assay use within the field. In this study, we have reported on the use of all techniques named in the characterisation of publicly available cell lines at UK and US repositories (NIH, ECACC, MRC, ATCC), whether these are sufficient to definitively determine pluripotency and the frequency with which the teratoma assay was used as part of this characterisation. As a consequence of our analysis, questions are raised about whether the teratoma xenograft assay still features as a key method in the characterisation human PSCs or if it has been superseded by other techniques. If so, do our observations guide us to the selection of a new ‘gold standard’ approach?

### Characterisation Data is not Routinely Published for ES Cell Populations

Initial assessment revealed that only 11% of cell lines did not have any characterisation data available, which seems relatively reasonable. However, the majority of these were embryonic (ES) cell lines, and it is concerning that almost half (41%) of the ES cell lines surveyed had no reported characterisation data. This may be due to the fact that such data were simply not published, which is possible if the lines had been derived intended for in-house use. However, this finding in itself raises separate issues not covered in this review on the requirement/responsibility of publishing cell line characterisation data, particularly if the cell line in question is eventually made publicly available.

### The Consistent, Concurrent use of Phenotypic and Functional Methods is Encouraging, with a Tendency Towards Simple, Rapid Techniques

The characterisation of putative human PSCs is crucial to ascertaining their identity as a true stem cell population. Many analyses focus on the pluripotent phenotype, providing a ‘snap shot’ assessment of cellular status through the detection of surface markers, expression of transcription factors and enzymes known to be present in PSCs. This is unsurprising, and likely due to the inexpensive nature of these techniques and the ease with which these analyses can be performed in almost any laboratory, without the need for highly specialist equipment. However, as has already been noted, pluripotent phenotype does not necessarily correlate with developmental potential, due to the fact that the mechanisms and signalling pathways which maintain pluripotency and ability to differentiate are distinct from those which execute lineage determination and differentiation [1]. Even powerful technologies such as single cell RNA sequencing, which can be used to quantitatively assess heterogeneity in pluripotent states and subpopulations within PSC lineages [55, 56], may not be able to detect these differences. Thus the use of additional methods is required to ascertain more thoroughly whether PSCs can successfully differentiate into mature, complex tissue structures representative of each germ layer.

It was encouraging to note that almost all cell lines examined in this study had at least one phenotypic and one functional assay performed. There is a trend towards simple, rapid methods such as immunocytochemistry and flow cytometry, which use a set of well-defined pluripotency markers as a basis for confirming pluripotency (see Table 1) [5]. As for functional assays, specific directed *in vitro* differentiation is highly favoured, which is a relatively straightforward method; it can be conducted within standard cell culture facilities and confirmed by differentiation marker expression. While the use of simple and rapid methods is less of an issue for general phenotypic assessments, such low demand techniques may neglect some of the finer aspects of functional characterisation, particularly the ability of cells to self-organise and achieve the functional maturity as seen in highly differentiated, organised tissue structures from mature teratoma xenografts.

### The Teratoma Assay is not Routinely Performed on iPSC Lines, Which may Result in an Incomplete Characterisation and Understanding of Cellular Behaviour

The teratoma xenograft assay has long been held as the definitive method to assess the differentiation capacity of human PSCs, and yet it was performed in only 13% of cell lines in this study, which seems at odds with its previous ‘gold standard’ status. Of the teratoma assays that were conducted, most were on ES cell lineages. In this study, we found that 72% of ES cell lines and only 0.9% of iPSC lines within the identified repositories had the assay performed. It is likely that this is due to the high-throughput nature by which new iPSC lineages are derived, rendering the assessment of developmental potential by a teratoma xenograft an unfeasible task if performed for each new line. There is no doubt that directed differentiation can provide a wealth of information regarding differentiation capacity, with some studies suggesting that *in vitro* differentiation could replace the teratoma assay for differentiation assessment [33, 87]. However, there is currently no standardised replacement for the teratoma assay which can definitively assess PSC differentiation and simultaneously provide information on malignancy. This is particularly relevant when we recall the functional heterogeneity between PSC populations expressing the same established pluripotency markers [1, 9], and that current reprogramming technologies are another source of potential variation for iPSCs. Incomplete reprogramming can lead to epigenetic anomalies, such as variability in X chromosome inactivation, insufficient silencing of source lineage DNA modifications and aberrant DNA methylation, all of which have the potential to impact on cellular behaviour, including differentiation capacity and phenotype stability in disease lines [88, 89]. While there seems to be consensus on the ability of the teratoma assay to provide malignancy data, recent studies have provided conflicting evidence regarding the ability of the assay to successfully identify incompletely reprogrammed iPSCs [33]. One study cites that these cells may form tumour masses similar to teratomas which do not contain the three developmental germ layers, and are open to misinterpretation [90]. On the one hand, the characterisation of these iPSCs could be considered incomplete due to a lack of teratoma assay to confirm differentiation capacity alongside more subtle aspects of cellular behaviour, such as malignancy. Yet conversely, the conflicting evidence on incompletely reprogrammed cells brings into question the suitability of the teratoma assay for assessing iPSC potency at all.

### High Variability in all Parameters and a Lack of Protocol Reporting Prevents Standardisation of the Teratoma Assay

Protocols used in the teratoma xenograft assay continue to demonstrate a high level of variability [12, 28]. Major parameters such as cell number, injection site and mouse strain differ considerably between studies, all of which are well known to impact on differentiation trajectory of PSCs, therefore affecting the tissues which form in the tumour [24–26, 72]. This is in addition to the natural biological variation inherent in such an assay through the use of an animal host. Such variation can be an issue when the xenograft assay is used to directly compare the differentiation capabilities of novel PSC lines, as if the microenvironmental cues provided to the cells on implantation are directly influencing their behaviour, the true differentiation capabilities and any subtle lineage biases or changes in potency may not be noted. This hampers reproducibility and leads to an inability to determine the suitability of a specific PSC line for an intended application, as well as difficulties in monitoring PSC performance over time for quality assurance. Overall, protocol analysis shows a lack of any commonalities that could be used to standardise the assay, with the modal response being that protocol data was not reported. The lack of transparency surrounding teratoma protocol reporting is not consistent with the expectations of rigour associated with robust scientific research.

The period of tumour growth and assay endpoint are other parameters which ultimately impact on the differentiation and maturity of tissues derived from the implanted PSCs. In part, this is dependent on whether the assay is stopped according to length of study, tumour dimensions, or factors concerning the welfare of the host. This is also variable and it is evident that teratomas which have been left to grow for longer will have a greater likelihood of forming larger, more complex and mature tissue structures. Choosing to end a teratoma assay based on a specific time frame at least allows for comparison within a study, but the same cannot be said if biological dimensions are employed, as biological variability in tumour growth could lead to significantly different xenograft growth periods. Regardless of when the endpoint is determined, the need for a full and detailed analysis of the resultant tumour is paramount. Assessment by a pathologist or other suitably trained personnel is recommended due to the presence of partially differentiated and immature structures [91]. Histological analysis should also be performed at separate sites within the tumour to account for variation across the tumour mass and avoid missing specific tissue structures. The way in which histological analysis was performed is not often provided in detail and the assessment of protocols examined herein. In this study, it was noted that a total of 22 cell lines included details that histological analysis had been performed by a trained pathologist (whether clinical, veterinarian or commercial).

The success rate of a teratoma xenograft study may also provide information regarding the health of the PSCs tested, protocol success and assay utility, as well as insights into the developmental potential of PSC lineages which have been genetically manipulated [22, 33, 92, 93]). Yet, only three cell lines out of all those surveyed provided success rate data; two ES cell lines (Nott-1 and Nott-2) which were derived in the same paper [94], and one iPS cell line (DXR0109B) [38]. The teratoma formation success rates were 60%, 100% and 25% respectively. This is a wide range and includes high failure rates, which may indicate the hesitancy of some to publish these data, given the inherent unreliability of the assay. The scarcity of success rate data also highlights a lack of clarity in reporting the number of animals used and therefore the number of biological or technical repeats performed. The regular omission of such key data seems at odds with the tight reporting regulations surrounding the use of animals.

### The Teratoma Xenograft Assay is not Often Performed, with Limitations and Technical Requirements Outweighing a Small Number of Strengths

The data presented herein and the recent study by Montilla-Roja et al. (2023), correlate strongly with that obtained around a decade ago [12]. While the proportion of ES cell lines with teratoma xenograft data appears to be higher (contrast 72% with Müller’s finding of 44%), differences in the datasets and relative numbers of each PSC type in the studies make it difficult to determine whether this is actually as a result of increased use of the assay. Similarly, and more recently, the difference in the number of iPSC lines which have had the assay performed is likely reflective of improved efficiency in derivation techniques, resulting in more lines and the inability to perform such an extensive characterisation.

As noted, only 13% of PSC lines in this study used the teratoma xenograft approach as a strategy to assess developmental potential. This may be due to a number of reasons including high experimental costs, need for appropriate licensing, labour intensive set up and inability to scale up, requirement for specialist technical skill and extended experimental time. The use of an animal host in a time when there is a sustained effort by the scientific community to find satisfactory alternatives to animal studies may well be the most influential factor in the lack of uptake. Ultimately, the lack of protocol information prevents replication and comparison, and while implementing measures such as minimum reporting standards could help remedy these issues (as has previously been seen with microarray studies and animal research [12, 86, 95]), the lack of standardisation remains evident. The International Society for Stem Cell Research (ISSCR) have recently produced a series of standards for the characterisation of human PSCs, which includes minimum analysis criteria, suggested analyses and minimum reporting standards for the various analyses, including the teratoma xenograft assay.

PluriTest has been suggested as an alternative method to assess pluripotency, given its ability to rapidly screen and compare to a large, evolving dataset comprised of well-characterised PSC lines. Concerns have been raised that bioinformatics methods such as this may not be able to provide sufficient information on malignant potential, a crucial aspect of pre-clinical safety assessment, whereas others reason that as data on differentiation defective or malignant cell lines is added to the database ‘PluriTest gains power to discriminate subtler characteristics of pluripotent cells’ [87]. This 2018 study by the International Stem Cell Initiative also noted that, while combining methods such as EB formation and PluriTest could provide a good overview of functional pluripotency, the final recommendation was that different analytical methods were required depending on the intended application [87]. As an ultimate application of many PSCs could be regenerative medicine, stringent methods are required to assess both malignant potential and differentiation potential in order to maintain patient safety. The teratoma xenograft assay is deemed to be the only assay currently able to assess the malignant potential of PSCs [28, 87], yet it has also been described as not having an acceptable level of reproducibility for iPSCs intended for the clinic [27] and there are concerns over the ability of the assay to identify incompletely reprogrammed cells. Accordingly, new approaches able to accurately and reproducibly assess both parameters are required.

### Innovative Three-dimensional Cell Culture Technologies Offer a Feasible Alternative to the Teratoma Assay

Although limited like all methods and models, *in vitro* studies by their nature are more reproducible and controllable, allowing for greater standardisation and comparability, and their greater throughput is also advantageous, enabling parallel studies to achieve large datasets. There have been recent developments where advancements in cell culture technologies have been applied to assess the developmental potential of PSCs *in vitro* and, in some cases, enabling the study of tissue formation and equalling the capability of the teratoma xenograft assay. The ability to form specific cell populations and complex single tissues from PSCs has been demonstrated by many, using varied techniques to create constructs similar to those *in vivo* [96–98]. Highly detailed culture protocols and expensive reagents/equipment can be prohibitive, and experiments such as these are perhaps too complex to use routinely in order to demonstrate the formation of tissue derivatives representative of all three germ layers. However, simpler methods which enhance the culture microenvironment to permit sufficient time for complex tissue structures to spontaneously develop have been used by various researchers to achieve the equivalency of the teratoma assay *in vitro*. Through using bioreactors to monitor culture conditions over long term studies, PSCs have been able to spontaneously produce teratoma like masses which are significantly more complex than EBs in a series of promising studies [81, 83, 99]. In our own laboratory, we combined EB formation and culture on a porous polystyrene scaffold (Fig. 5) to extend EB viability to the point where highly complex tissue structures can be clearly identified which show a very strong resemblance to those observed in teratoma xenografts (Fig. 6) [35, 37]. By allowing PSCs sufficient time to differentiate, similar to the bioreactor studies, PSC derived EBs were able to form diverse structures from the three germ layers, meeting the key criterion which underlines teratoma assay success. The controllability of this method, compatibility with varied analytical methods and ability to perform high throughput studies by culturing multiple EBs on the same scaffold are strong advantages, indicating the promising nature of this novel method for assessing pluripotent capacity. Some basic work regarding cellular malignancy and impaired differentiation capacity has been performed through the use of embryonal carcinoma cell populations in this system; these cells formed lineage restricted structures as expected and showed positive staining for pluripotency marker Oct4, indicating the presence of embryonal carcinoma elements in the tissue structure [37]. However, further optimisation is needed to fully explore these properties, using a wider range of differentiation defective and potentially malignant PSC populations, before this culture method can be considered a direct replacement for the teratoma assay.

The development and application of research tools such as these will allow researchers to achieve high levels of consistency and standardisation in the assessment of functional pluripotency. As such techniques become adopted over time, it is expected that the field will agree on a suite of new ‘gold standard’ methods and determine new criteria for an *in vitro* pluripotency assay, particularly one which enables the formation of recognisable tissue rudiments representative of all three germ layers and provides information on potential malignancy.

## Conclusions and Future Perspectives

In this article, we have discussed the methods used to characterise human PSCs, focusing in particular on the use of functional pluripotency testing to confirm differentiation potential. Through analysing the characterisation data of around 1500 PSC lines held at public cell banks/repositories, we have shown that while most had data available and had at least one functional assay performed, the classical approach using the ‘gold standard’ teratoma xenograft assay was only undertaken on 13% of cell lines in the study. The assay varies significantly in terms of protocols used and level of detail provided. This analysis significantly advances the conclusions of similar studies and concurs with the guidelines of major PSC institutions, such that standardising characterisation processes is essential to the progression and authenticity of PSC lineages used in the field, particularly in light of increased derivation of novel iPSCs, and PSCs use in clinical applications. We also note that standardisation of the xenograft approach has failed to materialise over time, with there being no clear replacement technique which can encompass the ability of the assay to assess differentiation capacity and malignant potential. As such, a new method must be sought to replace the previous ‘gold standard’ assay.

With the advent of modern culture technology and the need to reduce animal usage, we show how alternative *in vitro* approaches are now available that have the added benefit of achieving much of the same outcome as the original teratoma xenograft assay, focusing in particular on a method established within our laboratory using EBs and a porous scaffold. This method shows promise, forming structures which resemble those seen in teratoma tumours and fitting the success criteria, and could be optimised further to be considered a total direct replacement for the teratoma xenograft assay. As well as simply expanding the number of PSC lines used to gather valuable data on how different PSC populations behave within the *in vitro* system, perhaps leaning towards iPSCs given their rapidly expanding use, additional studies need to be conducted to assess differentiation defective PSCs, incompletely reprogrammed PSCs and potentially malignant PSCs. Direct comparison studies between PSCs would also be of benefit, as would quality monitoring studies on PSC populations over time, to ascertain whether the assay is powerful enough to discriminate subtle differences as a result of derivation/reprogramming method, disease status or genetic drift, and offer benefits above and beyond the teratoma assay, which has ultimately been hampered by a basic lack of standardisation.

Employing modern technology to enhance the growth environment of differentiating PSCs *in vitro* provides the opportunity to create improved strategies for assessing PSC function and their ability to form mature, organised tissue structures which may in time become a new ‘gold standard’ approach to assay potency. These methods have the potential to more readily provide essential information concerning the full characterisation of newly derived PSC cell lines, which in turn, will contribute to greater understanding of the developmental potential across different PSC lineages within the field, information that will be of significant benefit to the scientific community.

## Data Availability

The raw data supporting the conclusions of this article will be made available by the authors without undue reservation.
